# Supplementary material for: Complex‐centric proteome profiling by SEC‐SWATH‐MS
Source: Mol Syst Biol. 2019 Jan 14;15(1):e8438. doi: 10.15252/msb.20188438 (PMC6346213; doi:10.15252/msb.20188438)
Supplement: Supplementary file 8 — Dataset EV7 [file MSB-15-e8438-s008.zip › feature_plots_string/Q9BVA1.pdf]

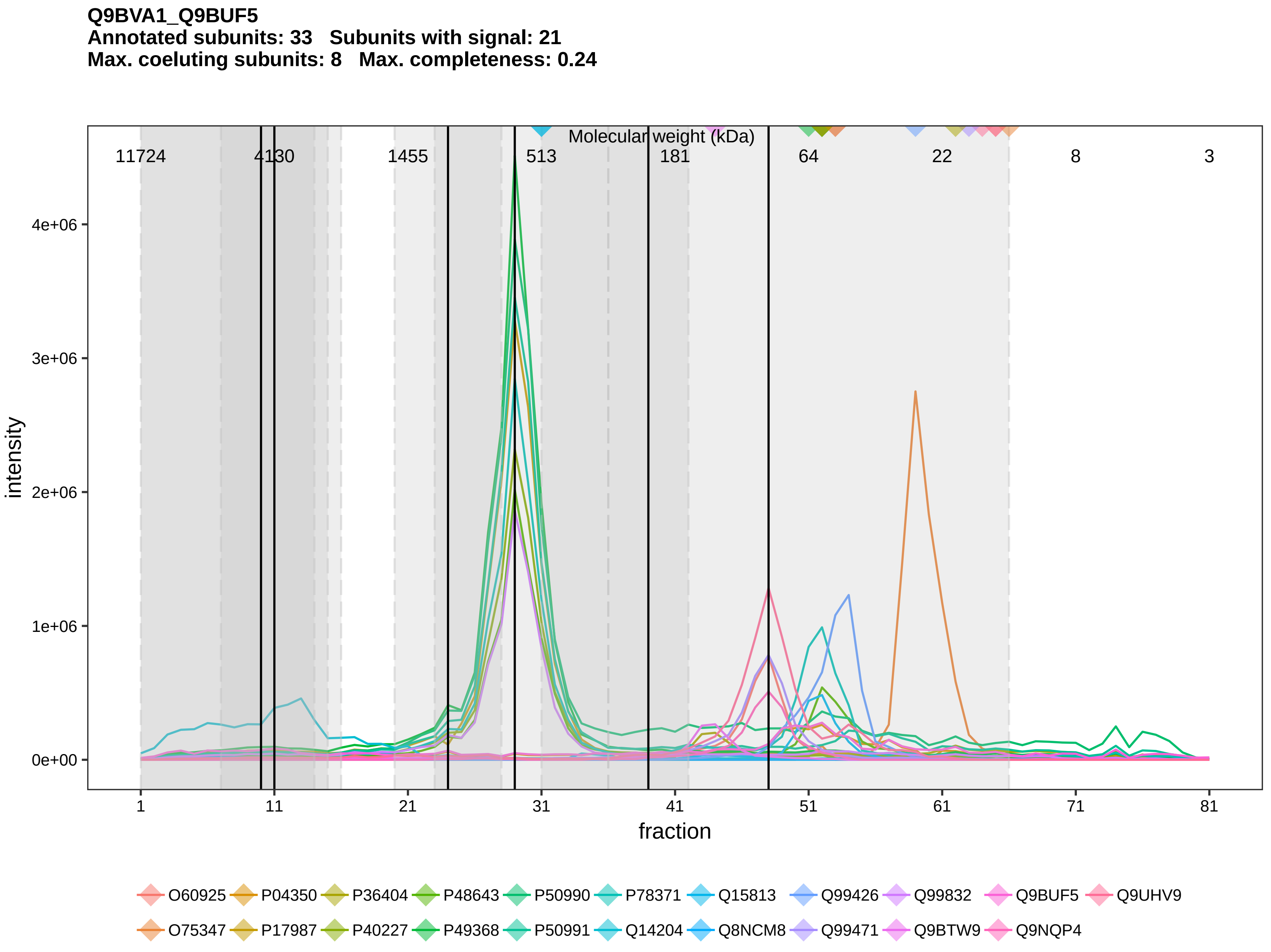
Q9BVA1_Q9BUF5
Annotated subunits: 33 Subunits with signal: 21
Max. coeluting subunits: 8 Max. completeness: 0.24
Molecular weight (kDa)
11724
4130
1455
513
181
64
22
8
3
intensity
4e+06
3e+06
2e+06
1e+06
0e+00
fraction
1
11
21
31
41
51
61
71
81
O60925
P04350
P36404
P48643
P50990
P78371
Q15813
Q99426
Q99832
Q9BUF5
Q9UHV9
O75347
P17987
P40227
P49368
P50991
Q14204
Q8NCM8
Q99471
Q9BTW9
Q9NQP4
